# A clinical test to assess isometric cervical strength in chronic whiplash associated disorder (WAD): a reliability study

**DOI:** 10.1186/s12891-022-05703-0

**Published:** 2022-08-01

**Authors:** Jeff Habberfield, Geoff Schneider, Kathryn Schneider, Sozina Katuli, Lee Olson

**Affiliations:** 1 Evidence Sport and Spinal Therapy, 2000 Veterans Place NW, T3B 4N2 Calgary, AB Canada; 2 Andrews University School of Rehabilitation Sciences. School of Rehabilitation Sciences Building 131, 8515 East Campus Circle Drive, 49104-0420 Berrien Springs, MI USA; 3 University of Calgary Cumming School of Medicine, 3330 Hospital Drive NW, T2N 4N1 Calgary, AB Canada; 4 Sport Injury Prevention Research Centre, Faculty of Kinesiology, University of Calgary, 2500 University Drive NW, T2N 1N4 Calgary, AB Canada; 5 Hotchkiss Brain Institute, University of Calgary, 2500 University Drive NW, T2N 1N4 Calgary, AB Canada; 6 Alberta Children’s Hospital Research Institute, University of Calgary, 2500 University Drive NW, T2N 1N4 Calgary, AB Canada

**Keywords:** Neck strength, Cervical strength, Whiplash associated disorder, Test–retest reliability, Test–retest agreement, Kinesiophobia, Neck disability

## Abstract

**Background:**

Cervical spine muscle weakness is well demonstrated in individuals with chronic neck pain. There is a lack of literature evaluating clinically applicable means of assessing isometric cervical strength in chronic whiplash associated disorder (WAD). This study assessed the reliability of self-resisted isometric cervical strength testing using a handheld dynamometer. The relationship between strength and neck pain-related disability and kinesiophobia was also investigated.

**Methods:**

Twenty subjects with chronic WAD performed maximum-effort isometric cervical flexion, extension, side flexion, and rotation against a hand held dynamometer. The dynamometer was held by the subject, who provided self-resistance. Subjects completed two sessions of testing on one day with two different examiners, and one session on a subsequent day with one of the original examiners. Subjects completed the Neck Disability Index (NDI) and Tampa Scale for Kinesiophobia (TSK) prior to the first testing session.

**Results:**

Intraclass correlation coefficients (ICC) for directional strength measures were fair to high (0.71–0.88 for intra-rater and 0.79–0.91 for inter-rater). Total strength (sum of all directional strengths) ICCs were high for both intra-rater (ICC = 0.91) and inter-rater (ICC = 0.94) measures. All statistical tests for ICCs demonstrated significance (α < 0.05). Agreement was assessed using Bland Altman (BA) analysis with 95% limits of agreement. BA analysis demonstrated difference scores between the two testing sessions that ranged from 3.0—17.3% and 4.5—28.5% of the mean score for intra and inter-rater measures, respectively. Most measures did not meet the a priori standard for agreement. A moderate to good inverse relationship was demonstrated between kinesiophobia (TSK score) and six out of seven strength measures (α < .05). No significant correlation was found between neck disability (NDI) and cervical strength in any direction.

**Conclusion:**

This study demonstrated fair to high reliability of self resisted isometric cervical strength testing in the chronic WAD population. All directional strength measures except flexion demonstrated a significant inverse relationship with kinesiophobia. No cervical strength measures were correlated with neck disability. These results support testing cervical strength in this manner to reliably assess change over time within individual patients. The value of such measurement requires further consideration given the lack of correlation between cervical strength and disability. Further research is required to establish normative values and enhance clinical utility.

**Supplementary Information:**

The online version contains supplementary material available at 10.1186/s12891-022-05703-0.

## Background

Whiplash associated disorders (WAD) have an annual incidence of at least 300 per 100,000 in the western world [1–4], and an often prolonged and complex recovery. Follow up data ranging from six months to two years post injury demonstrates that 40–60% of patients continue to report moderate to severe symptoms and disability [5–11]. Currently available diagnostic imaging is unable to clearly and consistently identify structural injuries as a definitive cause of symptoms following whiplash trauma [12]. As a result, many clinicians and researchers have shifted the focus to assessing function in these patients. In fact, most neck pain disorders are identified based on impaired function rather than demonstrable physical lesions [13, 14]. This involves assessment of both cervical muscle function (motor control) and strength (isometric strength). Impairment in deep cervical flexor motor control and endurance has been consistently identified in individuals with neck pain of both insidious and traumatic onset, including whiplash [15–20]. In addition to these changes in motor control, individuals with neck pain also demonstrate isometric weakness of the cervical muscles [21–25] compared to healthy controls, and non-recovery in WAD at one year is associated with such weakness [25].

The high prevalence of muscle dysfunction in chronic WAD dictates that it be properly assessed in order to identify its presence, guide treatment, and monitor change over time. While a reliable and valid means of assessing deep cervical flexor function has been established in the WAD population [15, 16, 18, 19, 26–28], no such measure exists for assessing general neck strength in a variety of movement planes in this patient group. The most standardized and objective means of assessing cervical strength is fixed-frame dynamometry (FFD), in which the load cell is fixed against a stable surface such as a wall or frame [29]. While highly objective, these devices are large, expensive, and impractical in most clinical settings.

It is well documented that individuals with neck pain lack the cervical strength of similar, healthy individuals [21–25]. Despite this a reliable, valid, and clinically practical means by which to assess it in all planes of motion does not exist. Although a correlation between isometric cervical strength and neck disability and function has not been identified in previous studies of large groups [23, 30, 31], it is unclear if individual changes in isometric strength over time are correlated with improved function. Therefore, reliable assessment of isometric strength has potential value in the assessment and treatment of these individuals. A tool that could adequately assess cervical strength in this way would help clinicians direct treatment to patient-specific areas of weakness, informing themselves and patients, and potentially improve outcome. It would also provide an objective means for assessing progress and change, which would be valuable to clinicians, researchers, patients, and third parties. One such measure, using self resistance with a handheld dynamometer, has been reported [32]. This method is proposed as a simplified, more clinically accessible approach to cervical strength measurement. For this assessment, the patient sits with the neck in a neutral position, holding a hand held dynamometer against the corresponding area of the head, and applies force in the desired direction of neck movement, using his or her arm(s) to provide counter pressure. The reliability of this method has been assessed for eight movements of the cervical spine (flexion, extension, side flexion bilaterally, combined side flexion and rotation bilaterally, and pure rotation bilaterally) in 30 healthy subjects [32]. Intra session reliability (ICC) ranged from 0.94-0.97, and inter session reliability, taken one week later, was 0.87-0.95. This established the test as reliable in a small sample of healthy subjects. It has, however, not been evaluated in patients with neck pain, which is the demographic it would be applied to in a clinical setting.

There were two primary aims of this reliability study. The first was to assess the reliability of self-resisted cervical strength testing in patients with chronic whiplash associated disorder (WAD). The primary research question for this aim was: what is the intra and inter-rater reliability of a clinical test of self-resisted isometric cervical strength in subjects with chronic whiplash associated disorder (WAD)? The hypothesis for this question was that the proposed clinical test demonstrates high intra and inter-rater reliability for measuring isometric cervical strength in subjects with chronic WAD. The second aim was to assess the relationship between self-resisted cervical strength, measured in this manner, and self-perceived neck disability and kinesiophobia. The research question was: what is the relationship between self-resisted isometric cervical strength and neck disability, and kinesiophobia, in chronic WAD subjects? The hypothesis for this question was that there is an association between self resisted isometric cervical strength and neck disability, and kinesiophobia, in chronic WAD subjects.

## Methods

### Setting

All testing took place at one multidisciplinary physical therapy and pain medicine clinic in Calgary, Alberta, Canada. Testing was conducted and overseen by two physiotherapist examiners with at least ten years of experience in outpatient orthopaedic practice and diplomas in orthopaedic manual therapy.

### Participants

Subjects were recruited from a multidisciplinary physical therapy and pain medicine clinic in Calgary, Alberta using consecutive sampling. The inclusion criteria were: a diagnosis of WAD grade 2 or 3 according to the Quebec Task Force on Whiplash Associated Disorders [33]; between 3 months and 6 years post injury; english speaking; age 18 to 65 years. Exclusion criteria were: pain with isometric contraction that prevented participation (subject unwilling to perform test due to pain), neuromuscular disorders, upper extremity injury preventing adequate range of motion or generation of counter force, and injury to the head preventing application of the hand held dynamometer.

A power analysis was performed to determine required sample size based on proposed power of 1-β = 0.8, α = 0.05, minimal acceptable reliability of ρ_0_ = 0.7, and desired reliability of ρ_1_ = 0.9. These numbers were inputted to an exact statistical calculation [34] and a validated approximation formula [35], yielding a required sample size of 19. Twenty subjects were included to allow for a 5% drop-out rate.

### Procedures

Following informed consent, subjects were assigned a subject number upon entering the study. Strength measures for the three testing sessions and outcome measures were kept in separate files. Testing occurred on two separate days, identified as “session A” and “session B”, within two weeks of each other. Prior to testing on the first day, one examiner (E1) conducted a brief upper extremity scan and reviewed the history to ensure the inclusion criteria were met. During session A, subjects completed the neck disability index (NDI) and Tampa Scale for Kinesiophobia (TSK).

Self-resisted isometric cervical strength was measured using a technique similar to that described by Versteegh et al. [32]. A calibrated MicroFET 2 hand held dynamometer (Hoggan Health Industries, Salt Lake City, Utah) was used to measure force. For all test positions, subjects were seated comfortably with feet flat on the floor, and knees and hips flexed to 90º (Appendix A). For flexion, the subject held the dynamometer with his or her dominant hand on the forehead. The other arm supported at the elbow of the dominant hand, with its elbow against the stomach. For extension, the pad was placed on the occiput with both hands holding the force pad for resistance. Side flexion was tested with the pad immediately above the ear, held by the ipsilateral hand, with the elbow supported against a wall. Rotation was tested with the pad placed on the temple and the elbow supported against a wall. The neck was in neutral anatomical position during for all test positions. Testing positions and standardized instruction are presented in Appendix A. Prior to testing, subjects familiarized themselves with each movement by practicing with a sub maximal contraction. Subjects were instructed to gradually build up force over three seconds, consistent with previous studies [13, 21, 32], ultimately pushing as hard as possible. One measurement was taken in each direction, consistent with the Versteegh study. Previous investigations of cervical strength have found that when multiple measures are taken in each direction, the first attempt consistently yields the highest value [13, 36]. Therefore, multiple attempts were deemed unnecessary. One attempt was performed in each direction. Subjects were given 30–60 s of rest between each test, consistent with previous studies investigating cervical strength [22]. Rolling of a die determined the order in which the movements were performed for each session. Observed force values for each direction, as displayed on the MicroFet 2 dynamometer in kgf, were recorded into the digital data file. After a ten minute break, testing was repeated under the supervision of the other investigator (E2).

Session B of testing occurred within two weeks of session A. This timeframe allowed for rest without enabling enough time for significant strength changes to occur, and it is similar to previous investigations of neck strength testing [22, 32]. During session B, subjects again performed the six self-resisted cervical movements, using the same protocol as during session A, supervised by E1. Session B was used to determine intra rater reliability.

Examiners and subjects were blinded to previous test results and outcome measure scores during testing, and data for each session was stored in separate documents. Data was not combined until all sessions were completed for all subjects. Session A and B did not necessarily occur in chronological order, depending on subject and examiner availability.

Study design was in accordance with the quality appraisal tool for studies of diagnostic reliability (QAREL) [37, 38]. Examiner bias was limited by the self-resisted nature of the strength testing. Subjects were given standardized instructions and examiners followed a specific protocol (see Appendix A). Observed strength values were measured using a calibrated digital dynamometer. The only examiner influence was on patient positioning and instructions, which were both standardized.

### Measurement and instrumentation

Self-reported neck disability was measured using the Neck Disability Index (NDI), a patient-reported outcome measure used to assess neck pain and disability [39]. The NDI consists of 10 items, each scored on a six point ordinal scale from zero to five, with higher scores representing greater disability. The NDI has been found to be a valid measure of neck function for patients with WAD as well as idiopathic neck pain [40], and it has demonstrated high internal consistency and validity in WAD patients [41].

Kinesiophobia was measured using the Tampa Scale for Kinesiophobia (TSK). Kinesiophobia (the fear of movement, injury, or reinjury [42]) is a psychological feature often investigated in WAD [7, 23, 43–45]. The TSK is a 17 item questionnaire, with each item scored on a 4 point ordinal scale. Total score ranges from 17 to 68, with higher scores representing greater kinesiophobia [42].

In the assessment of inter-rater and intra-rater reliability and agreement, the independent variables were the raters and testing session, and the dependent variables were cervical flexion, extension, right and left side flexion, right and left rotation, and total (sum of all directional measures) strength, measured in kgf. For secondary analysis, the dependant variable was neck strength (all directions listed above) and the independent / explanatory variables were neck disability (NDI) and kinesiophobia (TSK).

### Statistical analysis

Data analysis was performed using SPSS software version 26 (IBM, Armonk, New York). Descriptive statistics were computed to illustrate participants’ demographic characteristics. Shapiro–Wilk test was used to assess for normal distribution. Repeated measures ANOVA testing was conducted to determine if the order in which a direction was tested affected the relative strength of that direction (as a percentage of the total strength for that testing session).

The Pearson product-moment coefficient of correlation was used to determine the association between age, body weight, and directional strength values. This was also used to assess internal consistency (Cronbach’s α). Intra-rater and inter-rater reliability was analyzed for all directional strength and total strength values using intraclass correlation coefficient (2,1) [46–48]. To further assess reliability, standard error of measurement (SEM) was estimated from the standard deviation of the average scores for each testing sequence. The SEM is an estimate of the random variation in scores that would be expected when no actual change has occurred, expressed in the unit of measurement (in this case kgf) [49]. The minimum detectable change (MDC) can be calculated from the SEM. The MDC can be interpreted as the minimum amount of change that is required to consider that a real difference has occurred [49]. The MDC was estimated based on a 95% confidence interval of the SEM, which is equal to the SEM × 1.96 x √2.

Intra and inter-rater agreement, which provides information regarding the absolute difference in scores of a repeated test [50], was assessed via Bland–Altman (BA) analysis with 95% levels of agreement. BA analysis compares the scores of two measurements using difference scores and the mean and standard deviation of those difference scores [50]. The standard for acceptable agreement is subjectively determined a priori by the investigators based on the intended application and interpretation of the test [50, 51]. In this study, the a priori acceptable standard of agreement for each directional strength measure was based on the minimal detectable change found the previous study that investigated this form of strength measurement in healthy individuals [32]. If the total breadth of the 95% levels of agreement was less than the previously reported MDC for a given strength measure, it would be concluded that agreement was acceptable for that measure.

Correlation between each strength measure and neck disability (NDI score) and kinesiophobia (TSK score) was assessed with Pearson-product moment correlation for parametric data (TSK score) and Spearman rank correlation for non-parametric data (NDI score). Correlation analysis was performed using subject’s mean score for each directional measure. All comparisons were tested against α < 0.05. Correlation coefficients of less than 0.25 indicate little or no relationship; coefficients of 0.25-0.5 indicate a fair relationship; 0.5-0.75 indicate a moderate to good relationship; and coefficients greater than 0.75 indicate a good to excellent correlation [46]. Regression analysis was performed for outcome measures (TSK and/or NDI) that were found to have a significant correlation with total strength to quantify the relationship. The sample size was not adequately powered to assess correlation with sex-separated strength, and thus this was not performed. Correlations were assessed for both NDI (Spearman rank) and TSK (Pearson product-moment) with percentage of total strength values in each direction, to determine if relative strength or weakness of one directional movement was associated with NDI or TSK score. Regression analysis was performed for TSK on the outcome variable average total strength to assess for a relationship.

### Safety and ethics

There were no anticipated risks to the subjects in this study. No intervention or withholding of treatment was involved. In the previous study that used a similar protocol for assessing cervical strength, none of the 30 subjects reported any pain or discomfort during or after testing [32]. Other studies assessing maximal isometric cervical strength via fixed-frame dynamometry have reported no pain associated with testing in both healthy subjects and those with neck pain [13, 21, 22, 52, 53]. Neck soreness was acknowledged as a possible risk to subjects. Subjects were informed of the risk, and given the contact information for one of the physiotherapist researchers (E1) in case of any adverse reaction to the testing. Informed consent was obtained from all subjects, including for publication of identifying images. No adverse response to testing was reported. Ethics approval was granted by the University of Calgary (REB18-0851) and Andrews University (IRB 17–128). All methods were performed in accordance with relevant guidelines and regulations.

## Results

Subject demographics are presented in Table 1. All subjects had undergone at least four months of multimodal treatment (education, motor control exercises, postural strengthening, manual therapy) immediately prior to testing.

**Table 1:** Demographics of study sample

	**Female**	**Male**	**Total**
**N**	15	5	20
**Age**	36 (21–52)	40 (25–50)	37 (21–52)
**Weight**	165 (122–360)	187 (155–215)	170 (122–360)
**NDI**	17.7 (7–30)	17.6 (9–44)	17.7 (7–44)
**TSK**	37.7 (30–51)	38.8 (23–60)	38 (23–60)
**Duration**	21.5 (6–70)	15.6 (4–26)	20 (4–70)

Values are mean scores (range in brackets). Age (years); weight (pounds), *NDI* Neck disability index score, *TSK* Tampa scale for kinesiophobia score, *Duration* Months since accident

### Strength measures

Average strength values are presented in Table 2. All measures were normally distributed. Strength values separated by sex are displayed in Table 3. One male subject demonstrated markedly decreased strength (all directions less than 1 kgf), which altered the mean values in the small sample group of five males. In all directions male strength values were greater than female, and this difference increased when the data of the outlier male was removed. This male outlier had the shortest duration of WAD symptoms of all subjects (4 months post injury), and had the highest TSK score (60/68) of all subjects. Sequence of testing was not found to have any significant influence on the relative strength of any movement direction.

**Table 2:** Average strength values (kgf) of the total sample (*n* = 20)

	**Average**	**Range**	**Std. dev**	**% of total**
**Flex**	3.15	.27—7.47	1.57	13.4
**Ext**	6.68	.5—14.2	3.52	27.4
**RSF**	3.70	.5—6.73	1.74	15.8
**LSF**	4.22	.4—8.97	2.28	17.4
**R Rot**	3.11	.27—5.97	1.52	13
**L Rot**	3.15	.3—5.93	1.55	13.1
**Total**	24.02	2.2—42.8	11.55	100

*Flex* Flexion, *Ext* Extension, *RSF* Rght side flexion, *LSF* Left side flexion, *R Rot* Right rotation, *L Rot* Left rotation, *Total* Sum of all directional strength values, % of *total* directional strength as a percentage of total strength

**Table 3:** Sex specific strength values

	***n***	**Flex**	**Ext**	**RSF**	**LSF**	**RR**	**LR**	**Total**
**Female**	15	2.97	6.53	3.61	3.84	2.97	2.97	22.9
**Male (all)**	5	3.69	7.13	3.99	5.37	3.51	3.69	27.38
**Male (outlier removed)**	4	4.54	8.79	4.87	6.62	4.32	4.53	33.67

*N* Sample size, *Flex* Flexion, *Ext* Extension, *RSF* Right side flexion, *LSF* Left side flexion, *RR* Right rotation, *LR* Left rotation, *Total* Sum of all directional strength values, male (outlier removed) average male data after removal of outlier subject that demonstrated significant weakness

Neither body weight nor age was correlated with any directional or total strength measure (Table 4). All directional and total strength measures demonstrated internal consistency (α < 0.01), with Cronbach α values ranging from 0.78 to 0.97(Table 5).

**Table 4:** Correlation between age, weight, and directional neck strength measures (*n* = 20)

		**Flex**	**Ext**	**RSF**	**LSF**	**RR**	**LR**	**Total**
**Weight**	**R**	0.072	0.142	0.100	0.156	0.130	0.188	0.141
	***P*-value**	0.764	0.550	0.676	0.511	0.586	0.427	0.553
**Age**	**R**	0.096	0.068	0.178	0.191	0.249	0.147	0.151
	***P*-value**	0.686	0.777	0.453	0.421	0.289	0.537	0.526

*Flex* Flexion, *Ext* Extension, *RSF* Right side flexion, *LSF* Left side flexion, *RR* Right rotation, *LR* Left rotation, *Tota*l Sum of all directional strength values, *r* Pearson product-moment coefficient of correlation

**Table 5:** Internal consistency (Cronbach’s α) between strength measures (*n* = 20)

	**Flex**	**Ext**	**RSF**	**LSF**	**RR**	**LR**	**Total**
Flex	1	.796^a^	0.780^a^	0.836^a^	.847^a^	.824^a^	.883^a^
Ext	.796^a^	1	.934^a^	0.864^a^	.843^a^	.880^a^	.954^a^
RSF	.780^a^	.934^a^	1	0.919^a^	.920^a^	0.923^a^	.968^a^
LSF	.836^a^	.864^a^	.919^a^	1	.908^a^	.947^a^	.960^a^
RR	.847^a^	.843^a^	.920^a^	.908^a^	1	.920^a^	.945^a^
LR	.824^a^	.880^a^	.923^a^	.947^a^	.920^a^	1	.962^a^

*Flex* Flexion, *Ext* Extension, *RSF* Right side flexion, *LSF* Left side flexion, *RR* Right rotation, *LR* Left rotation, Total Sum of all directional strength values

^a^ all Cronbach α values significant (α < 0.01)

### Reliability

Intraclass correlation coefficient (ICC) values are displayed in Table 6. ICC for directional strength measures ranged from 0.713 to 0.882 for intra-rater reliability, and from 0.793 to 0.911 for inter-rater reliability. In both cases, the lowest correlation was for left side flexion, and the highest value was for right rotation. Total strength (sum of all strength values) ICC was 0.908 and 0.937 for intra-rater and inter-rater measures, respectively.

**Table 6:** Reliability of neck strength testing (*n* = 20)

		**ICC**	**95% CI**	**SEM**	**MDC**	**MDC %**
**Intra rater**	**Flex**	0.871	.673—.949	0.393	1.09	35.83
	**Ext**	0.830	.570—.933	1.071	2.97	47.42
	**RSF**	0.843	.603—.938	0.526	1.46	40.95
	**LSF**	0.713	.276—.887	1.367	3.79	89.37
	**RR**	0.882	.702—.953	0.349	0.97	33.13
	**LR**	0.814	.530—.926	0.556	1.54	51.03
	**Total**	0.908	.769—.964	1.973	5.47	23.74
**Inter rater**	**Flex**	0.870	.670—.948	0.453	1.26	40.50
	**Ext**	0.904	.758—.962	0.722	2.00	30.23
	**RSF**	0.868	.665—.948	0.443	1.23	33.64
	**LSF**	0.793	.476—.918	0.995	2.76	67.43
	**RR**	0.911	.776—.965	0.267	0.74	24.27
	**LR**	0.853	.628—.942	0.471	1.31	40.17
	**Total**	0.937	.841—.975	1.496	4.15	17.46

*ICC* Intraclass correlation coefficient, 95% *CI* 95% confidence interval of *ICC*, *SEM* Standard error of measurement, in kgf, *MDC* Minimum detectable change, in kgf, *MDC* %, *MDC* as percentage of mean strength score for given direction for all subjects, *Flex* Flexion, *Ext* Extension, *RSF* Right side flexion, *LSF* Left side flexion, *RR* Right rotation, *LR* Left rotation, *Total* Sum of all directional strength values

### Agreement

Agreement was assessed using Bland–Altman (BA) analysis with 95% limits of agreement. These were compared to the a priori standards for acceptable agreement, based on previously reported MDC values [32]. A Measure was deemed to show agreement if the total breadth of the 95% limits of agreement was less than the MDC value. Only intra-rater flexion and intra-rater total strength measures met the standard for acceptable agreement. Comprehensive results of the BA analysis are displayed in Table 7.

**Table 7:** Assessment of each strength measure for acceptable agreement (*n* = 20)

		**Mean score**	**Difference Scores**					**Difference Scores as % of Mean Score**		
			**Mean**	**St. Dev**	**95% Limits**			**% of Mean**	**95% Limits**	
					**Lower**	**Upper**	**A priori std**		**Lower**	**Upper**
**Intra rater**	**Flex**	3.04	0.415	1.094	-1.73	2.56	5.17^a^	13.65	-56.89	84.19
	**Ext**	6.26	-0.185	2.597	-5.27	4.90	5.64	-2.96	-84.26	78.35
	**RSF**	3.56	0.615	1.328	-1.99	3.22	4.08	17.28	-55.84	90.39
	**LSF**	4.24	0.460	2.552	-4.54	5.46	3.38	10.85	-107.11	128.80
	**RR**	2.92	0.125	1.017	-1.87	2.12	3.57	4.28	-63.99	72.56
	**LR**	3.02	-0.160	1.289	-2.69	2.37	4.29	-5.30	-88.93	78.33
	**Total**	23.04	1.270	6.504	-11.48	14.02	26.13^a^	5.51	-49.82	60.84
**Inter rater**	**Flex**	3.10	-0.545	1.255	-3.00	1.91	3.13	-17.58	-96.93	61.77
	**Ext**	6.62	-1.815	2.331	-6.38	2.75	4.72	-27.42	-96.42	41.59
	**RSF**	3.65	-0.690	1.218	-3.08	1.70	3.65	-18.90	-84.32	46.51
	**LSF**	4.09	-0.185	2.187	-4.47	4.10	3.07	-4.52	-109.32	100.28
	**RR**	3.05	-0.870	0.894	-2.62	0.88	2.66	-28.52	-85.97	28.92
	**LR**	3.25	-0.360	1.229	-2.77	2.05	3.17	-11.08	-85.21	63.05
	**Total**	23.75	-4.465	5.962	-16.15	7.22	20.40	-18.80	-68.00	30.40

*Mean score* mean strength score, *mean* mean difference score between two measures (measure 1—measure2), St. *Dev* standard deviation of difference scores, A priori* std* previously reported *MDC* values for each strength measure, used as threshold to assess for agreement; % *mean* mean difference score as percentage of mean strength score, *Flex* Flexion, *Ext* Extension, *RSF* Right side flexion, *LSF* Left side flexion, *RR* Right rotation, *LR* Left rotation, *Total* sum of all directional strength values

^a^ Acceptable agreement observed if breadth of 95% limits of agreement less than a priori std

All strength values expressed in kgf

### Outcome measures and correlation with strength

NDI scores were not normally distributed (Shapiro–Wilk sig. = 0.03), with a mean score of 17.65 (range 7–44). TSK scores were normally distributed with a mean score of 37.95 (SD 8.09). NDI was not correlated with any directional or total strength measure or TSK score (Table 8), as determined via Spearman rank correlation. All directional measures and total strength demonstrated a significant, moderate to good inverse correlation with kinesiophobia except for flexion (Table 8).

**Table 8:** Correlation between NDI, TSK, and strength measures (*n* = 20)

		**TSK**	**Flex**	**Ext**	**RSF**	**LSF**	**RR**	**LR**	**Tot**
**NDI**	**Spearman**	0.169	-0.320	-0.239	-0.245	-0.256	-0.219	-0.183	-0.205
	**Sig**	0.477	0.169	0.309	0.297	0.276	0.354	-0.441	0.387
**TSK**	**Pearson**	1	-0.352	-0.567^*^	-0.623^*^	-0.523^*^	-0.565^*^	-0.548^*^	-0.566^*^
	**Sig**		0.128	0.009	0.003	0.018	0.009	0.012	0.009

*NDI* Neck disability index rank, *TSK* Tampa scale for kinesiophobia score, *Pearson* Pearson product-moment coefficient of correlation (*r*), with significance, *Spearman* Spearman rank correlation (r_s_) with significance, *Flex* Flexion, *Ext* Extension, *RSF* Right side flexion, *LSF* Left side flexion, *RR* Right rotation, *LR* Left rotation, *Total* sum of all directional strength values.All strength values expressed in kgf

^*^ significant correlation (*p* < .05)

No significant correlations were identified between NDI (Spearman rank) and TSK (Pearson product-moment) with percentage of total strength value of each direction, indicating no correlation with any relative directional weakness.

Regression analysis for TSK on the outcome variable average total strength identified a linear relationship (*p* = 0.005). Normal distribution of residuals was clearly apparent on a normal P-P plot, and homoscedasticity was visible as random distribution on a scatter plot. The adjusted r^2^ value for TSK and total neck strength was 0.282 (*p* = 0.009). That is, 28.2% of the total variation in total neck strength was predicted by TSK. In this linear model using TSK to predict total neck strength, a significant coefficient of -0.808 was found, suggesting that for every one point increase in TSK score, total neck strength score decreased by 0.808 kgf.

## Discussion

### Intra-rater and inter-rater measurements—reliability and agreement

In this study of self-resisted cervical strength testing in subjects with WAD, both reliability and agreement must be considered. Reliability indicates the consistency or reproducibility of a measure, while agreement is the degree to which repeated testing produces similar scores on subsequent testing of the same measure [49, 54–56].

For the assessment of reliability and interclass correlation coefficient (ICC), the guideline proposed by Meyers and Blesh [57] was applied. This states that an ICC below 0.69 is ‘poor’, 0.7 to 0.79 is ‘fair’, 0.8 to 0.89 is ‘good’, and above 0.9 is ‘high’. By this classification, intra-rater and inter-rater reliability was ‘fair’ for intra and inter-rater left side flexion; ‘high’ for inter-rater extension, right rotation, and both total scores; and ‘good’ for all other measures. Total strength reliability values were 0.908 and 0.937 for intra-rater and inter-rater, respectively. This is similar to previously reported reliability for fixed frame dynamometry, which ranges from 0.80 to 0.99 [21, 23, 36, 52, 53, 58]. The study by Versteegh et al. [32], which also used self resisted dynamometry, reported intra-session reliability of 0.94 to 0.97, and inter-session reliability of 0.87 to 0.95.

Minimal detectable change (MDC), which is the amount of change required to be confident that a real change has occurred [49], ranged from 0.97 to 3.79 kgf for intra-rater measures, and 0.74 to 2.76 kgf for inter-rater. The MDC for total strength values was 5.47 kgf for intra-rater, and 4.15 kgf for inter-rater. These values are approximately one third to one half of those found in the Versteegh study [32]. Mean strength values in the Versteegh study were 3.5 to 4.5 times stronger than in this study, suggesting that the SEM and MDC do not increase linearly with strength, and greater relative change in strength is needed at lower strength levels to indicate real change.

Agreement was assessed using Bland–Altman (BA) analysis with 95% limits of agreement. Only two of the measures, intra-rater flexion and total strength, were determined to show agreement based on the a priori standard. However, interpretation of agreement requires additional context given the markedly low values observed in this study. There are no standardized guidelines for acceptable agreement values [50]; rather, the value is judged based on its proposed use and the variable being tested. Prior to data collection in this study, previously reported minimal detectable change (MDC) values [32] were selected as a threshold against which the 95% limits of agreement would be measured. At the time of selecting this a priori threshold, it was not anticipated that subjects would demonstrate such profound weakness. This weakness offers context to the agreement analysis and its interpretation. For example, the average difference score (difference between two measurements) for intra-rater flexion was 0.415 kgf (Table 7). This represented 13.65% of the mean flexion score between the two intra-rater measurements, which was 3.04 kgf. In this case, a 13.65% difference would be reasonable for measures of isometric strength testing. However, this value only represents the mean difference, and does not inform on the possible range of differences. To improve interpretation, 95% confidence intervals (or limits of agreement) are used. In the example of intra-rater flexion, the 95% confidence interval, expressed as a percentage of mean score, was -56.89% to 84.19%. This means that 95% of difference scores will vary between 56.89% below and 84.19% above the original score when no real change exists. For all testing directions in this study, the mean difference scores ranged from -28.52 to 17.28% of the mean strength score. The confidence interval breadths ranged from 98.4 to 235.8% of the mean score. While this large range appears unacceptable, such relative values are inflated by the markedly low strength scores in this population. WAD subjects in this study demonstrated 20–30% of the strength of healthy subjects that were assessed in a very similar fashion in another study [32]. This must be considered when assessing clinical utility. Conservatively using the largest possible disagreement breadth (235.8% for intra rater left side flexion) dictates that a change of 117.9% in either direction is required to be 95% confident that a true change has occurred. While this appears onerous, such change is plausible, and even relatively modest, considering that subjects in this study would need to increase strength by a factor of 3.5 to 4.5 to reach normal levels [32].

When evaluating the difference scores, a negative bias was observed for all inter-rater measures, indicating higher average scores on the second testing session. This bias ranged from 0.185 to 1.815 kgf, or 4.52 to 28.52% of the mean strength score. This contradicts previous studies of isometric neck strength testing, which reported that the first attempt consistently yielded the greatest force when multiple attempts were allowed [13, 36]. These previous studies performed repeated contractions consecutively, in which the learning effect may have been negated by cumulative fatigue. The current study differed in that all directions were tested first, and then repeated after a 10 min break. Such a break would have allowed for recovery, and perhaps the increased score on the second round of testing reflected improvement associated with a learning effect or familiarity with the testing procedure. No such trend of positive or negative bias was noted for intra-rater measures.

This method of testing isometric cervical strength is clearly reliable in the chronic WAD population. The agreement analysis revealed a large range of difference scores for each measure. The ramification of this is not that the test is unusable; rather, it suggests that a large change must be observed between scores to be confident that a real change has occurred. It may seem appropriate to use the minimal detectable change (MDC) value to determine the extent of improvement required between sessions, however the MDC values are based on reliability and do not reflect the agreement of a repeated test. It would be novel, and more stringent in this case, to consider the Bland–Altman limits of agreement to determine if a real change has taken place. This may be termed “BA required change”. For example, the 95% confidence interval of difference scores for intra-rater flexion had a total breadth of 4.29 kgf, or 2.15 kgf in either direction. Consequently, an improvement of 2.15 kgf would be required to be 95% confident that the improvement was real and not due to measurement error. Measured this way, the BA required change for total strength would be 12.8 kgf for intra-rater and 11.7 kgf for inter-rater measurements. According to the MDC, these values would be 5.5 and 4.2 kgf, a much lower standard. BA required change values are presented in Table 9, along with the MDC values for comparison.

**Table 9:** Required change and minimum detectable change scores for each testing sequence (*n* = 20)

	**Rater**	**Mean score**	**BA Req Change**	**MDC**	**Healthy Score (kgf)**	
					**Female**	**Male**
**Flex**	Intra	3.04	2.15	1.09	10.7	21.3
	Inter	3.10	2.46			
**Ext**	Intra	6.26	5.09	2.97	19.45	33.85
	Inter	6.62	4.57			
**RSF**	Intra	3.56	2.61	1.46	11.35	22.05
	Inter	3.65	2.39			
**LSF**	Intra	4.24	5	3.79	11.25	22.2
	Inter	4.09	4.29			
**RR**	Intra	2.92	2	0.97	9.1	16.5
	Inter	3.05	1.75			
**LR**	Intra	3.02	2.53	1.54	8.5	15.7
	Inter	3.25	2.41			
**Total**	Intra	23.04	12.25	5.47	70.35	131.6
	Inter	23.75	11.69			

*Flex* Flexion, *Ext* Extension, *RSF* Right side flexion, *LSF* Left side flexion, *RR* Right rotation, *LR* Left rotation, *Total* sum of all directional strength values; *mean score* mean strength score between two rating, *BA Req Change* Required changed based on 95% confidence intervals of BA analysis (see description in text), *MDC* Minimum detectable change, *Healthy Score* strength measures from previous study of healthy subjects. All strength values expressed in kgf

### Association between strength, Neck disability, and kinesiophobia

Self-perceived neck disability, as measured by the Neck Disability Index (NDI), was not found to be significantly related to any strength measure. Previous studies have clearly demonstrated that individuals with neck pain are significantly weaker than those without [21, 30, 52, 59, 60], but the extent of weakness does not necessarily correlate with NDI score [23, 30, 31]. It has not been determined if individual changes in isometric strength over time are correlated with changes in function or disability. Establishing a reliable means of assessing strength enables this to be investigated. It is acknowledged that NDI scores were not normally distributed in this sample.

Six of the seven strength measures (all except flexion) demonstrated a significant inverse correlation with kinesiophobia, as measured using the TSK. It is therefore concluded that, in general, neck strength measured in this fashion is correlated with kinesiophobia. This may explain the extremely low strength scores in the male outlier with the highest TSK score of all subjects. When regression analysis was performed, kinesiophobia was the only variable found to be a significant predictor of strength, accounting for 28.2% of the variability in total cervical strength. This is consistent with previous reports that high scores of kinesiophobia are associated with decreased spinal performance [61, 62], but it differs from previous research suggesting no such relationship existed in WAD [23].

### Additional correlations

In this study of self-resisted isometric strength testing in subjects with WAD, neither weight nor age was found to be correlated with any directional neck strength value or total strength, consistent with previous studies in healthy subjects [36, 52, 53, 63]. This indicates that self-resisted neck strength testing does not require additional considerations or calculations for weight or age. When evaluating relative directional strength, extension accounted for the largest proportion of total strength at 27.4%, while the contribution of the other five directions ranged from 13 to 17.4%. No significant correlation was found between any of the relative directional strengths and neck disability (NDI) or kinesiophobia (TSK). The order in which each direction was tested was randomized for each testing session, and post hoc analysis found no effect of testing sequence on relative strength values for any direction of movement. In future studies and in clinical practice, randomization of testing sequence therefore appears unnecessary, and testing can be done in the most convenient order.

### Limitations

Neck pain intensity was not measured before and after every testing session. Intra-subject differences in neck pain intensity between testing sessions may have influenced force production. This study was powered to perform statistical tests on the entire sample. It was not adequately powered to assess sex differences or relationships with cervical strength separated by sex. Since females are known to be 40–50% weaker than males in isometric neck strength [29, 30, 32, 36, 52, 63], any correlations with neck strength will be influenced by the gender distribution within a sample. Some discrepancy in sex distribution is expected in studies of WAD, as there is modest evidence suggesting that women are more likely to experience chronic pain and a poorer outcome following whiplash than males [3, 45].

Additionally, the assessment technique examined here has not been validated against a gold standard. The gold standard for isometric neck strength testing would be fixed frame dynamometry. Validation was beyond the scope of this project, and this is an area for future research.

## Conclusion

Assessment of WAD focuses on clinical presentation and function, including motor function and strength [8, 15–21, 23, 52, 58, 64]. Despite known weakness in individuals with WAD compared to those without neck pain, there are no established clinically applicable means by which isometric cervical strength can be assessed. The current study evaluated a method of assessing self-resisted isometric cervical strength using a handheld dynamometer. This method was found to have fair to high intra-rater (ICC 0.713—0.882 for directional measures, ICC 0.908 for total strength) and inter-rater (ICC 0.793—0.911 for directional measures, 0.937 for total strength) reliability, supporting its clinical use. Agreement, which indicates the accuracy of the measure within an individual, was assessed with Bland Altman (BA) 95% limits of agreement. BA analysis demonstrated difference scores between the two testing sessions ranged from 3.0—17.3% and 4.5—28.5% of the mean score for intra and inter-rater measures, respectively. Subjects in this study demonstrated marked cervical weakness compared to healthy subjects tested similarly in a previous study [32].

A significant, moderate to good inverse relationship was demonstrated between TSK score and six of the seven strength measures. No significant relationship was demonstrated between any cervical strength measure and neck disability (NDI). Further investigation is warranted to confirm findings in a larger sample, establish normative values in the healthy population, determine if intra-subject changes in isometric strength are related to changes in function, and validate this technique against a gold standard such as fixed frame dynamometry.

## Supplementary Information

**Additional file 1: Appendix A. **Testing Protocol and Instructions.

**Additional file 2: Appendix B. **Bland-Altman plots with 95% confidence intervals of agreement.

**Additional file 3.**

## Data Availability

All subjects signed a consent form detailing the purpose of the study, procedure, and anticipated risks and benefits. The complete data set for this study can be obtained on reasonable request to the corresponding author (Jeff Habberfield, jhabberfield@gmail.com).
