# Supplementary material for: A clinical test to assess isometric cervical strength in chronic whiplash associated disorder (WAD): a reliability study
Source: BMC Musculoskelet Disord. 2022 Aug 1;23:736. doi: 10.1186/s12891-022-05703-0 (PMC9341054; doi:10.1186/s12891-022-05703-0)
Supplement: Supplementary file 1 — Additional file 1: Appendix A. Testing Protocol and Instructions. [file 12891_2022_5703_MOESM1_ESM.docx]

**Appendix A - Testing Protocol and Instructions**

Subjects are seated on a stool with hips and knees flexed 90º and the trunk and neck in a neutral position for testing directions. Rolling of a die will determine the order in which the movements will be performed for each session (1: flexion, 2: extension, 3: right side flexion, 4: left side flexion, 5: right rotation, 6: left rotation).

Prior to testing: “We are going to test your neck strength by seeing how hard you can push your head in six different directions. This device (MicroFET 2) will be used to measure how hard you push. For each direction, push with increasing force over a period of 2-3 seconds, building up to your maximal force. Try to keep the device still and push only with your head. Try not to push back with your arm. This should not cause a significant increase in pain. If it does, let me know.”

Before testing maximal strength in each direction, subjects may practice with a sub-maximal force
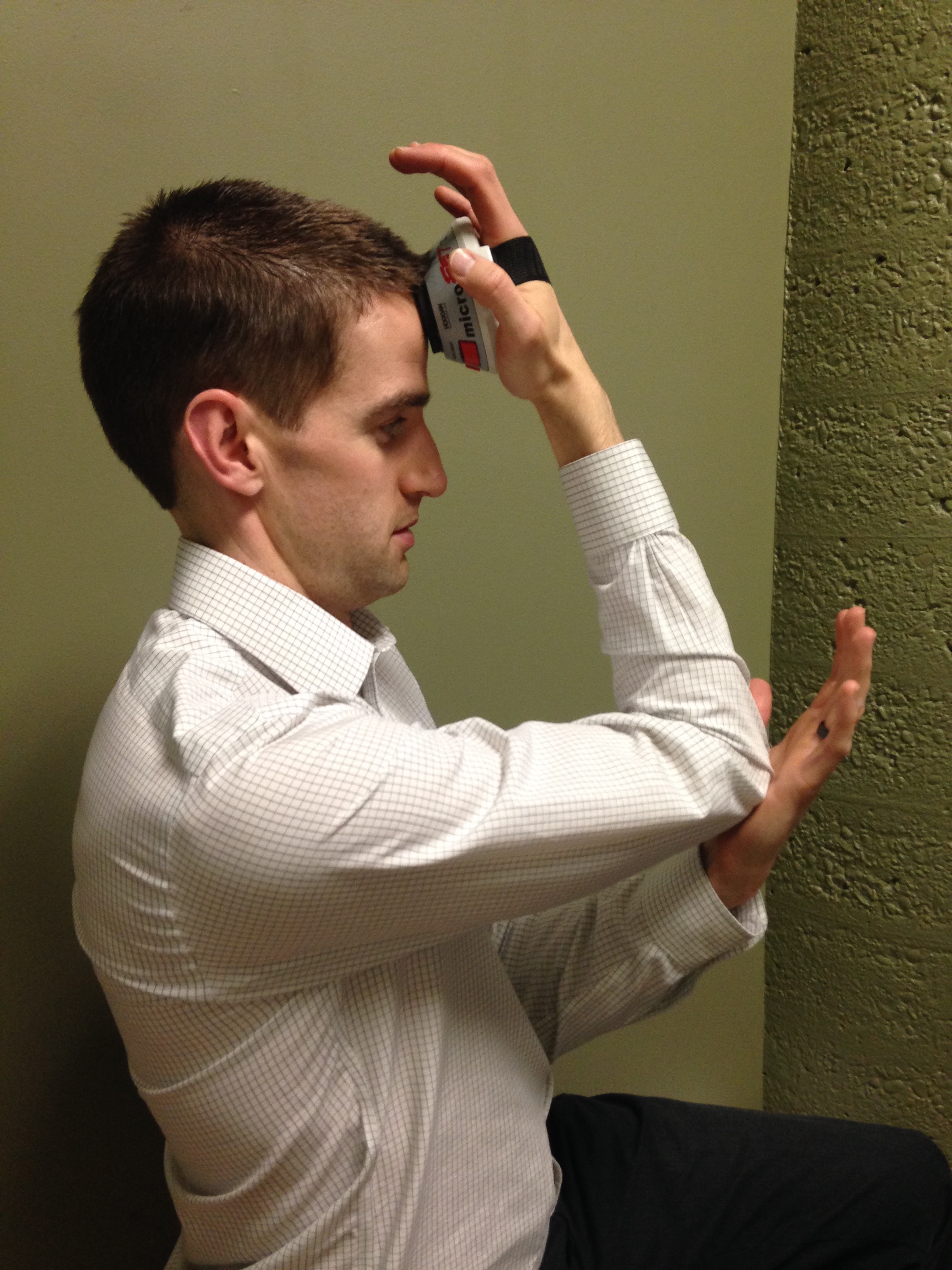
 to become familiar with the procedure. One attempt will be performed in each direction.

**Flexion**: the force pad is placed at the center of the forehead, supported by the palm of the dominant hand. The non dominant hand supports the elbow of the resisting arm, with the non dominant elbow against the stomach. “*Push forward with your head, as if you were trying to tip your head down to your chest*.”


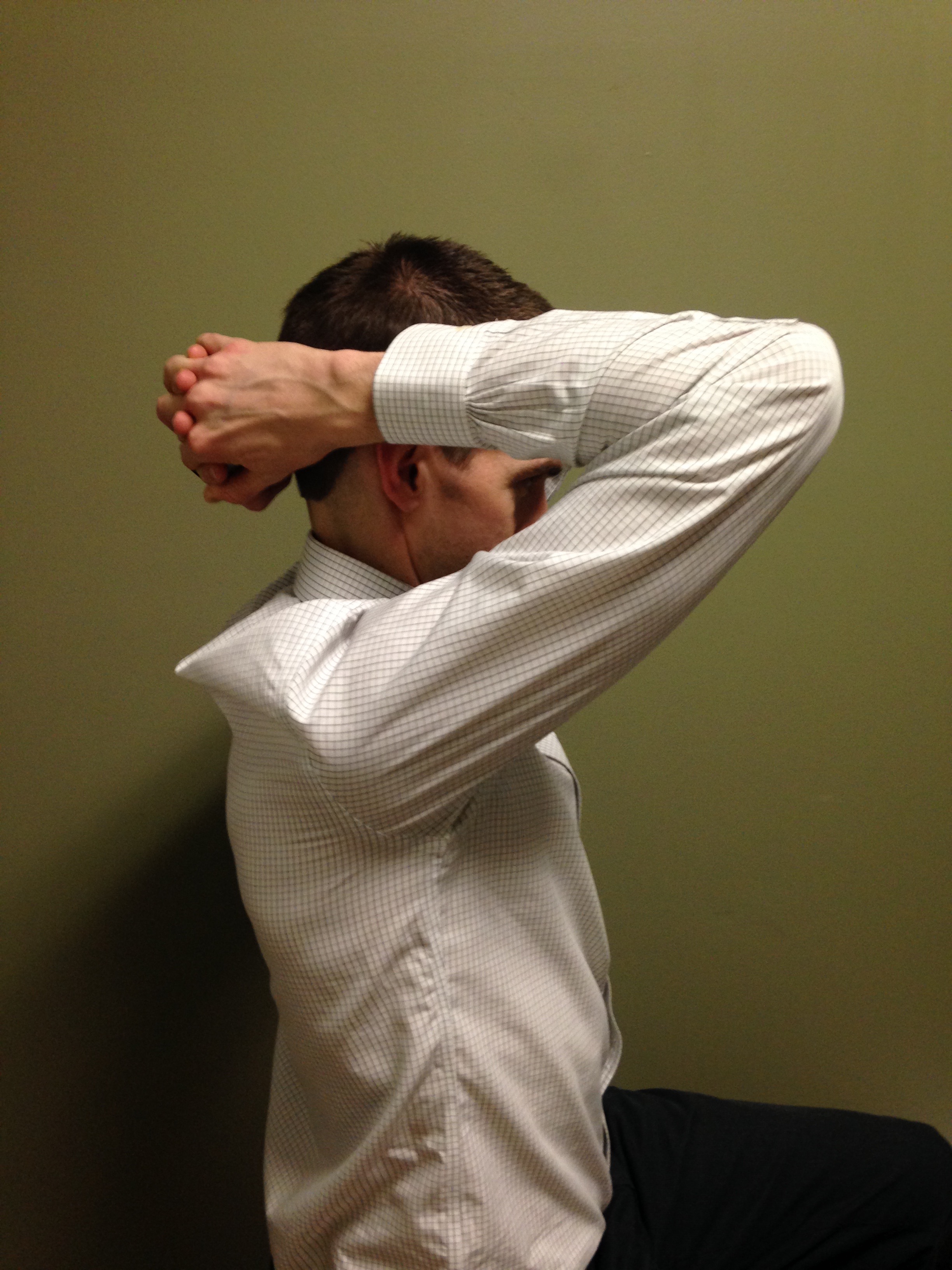


**Extension**: the force pad is placed over the external occipital protuberance. The dynamometer is held by both hands, with the fingers interlocked. “*Push backward, as if you were trying to tip your head back*.”


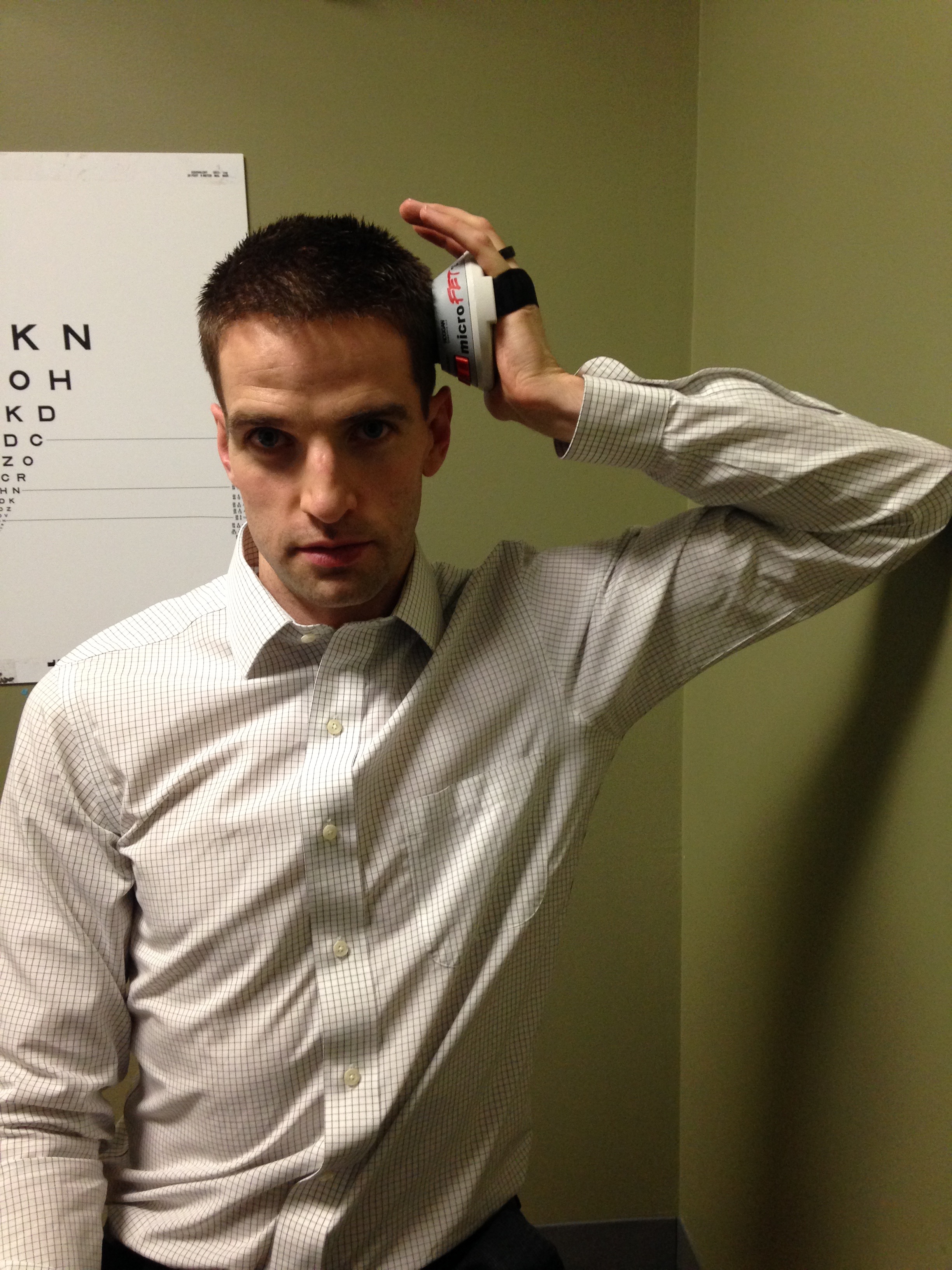


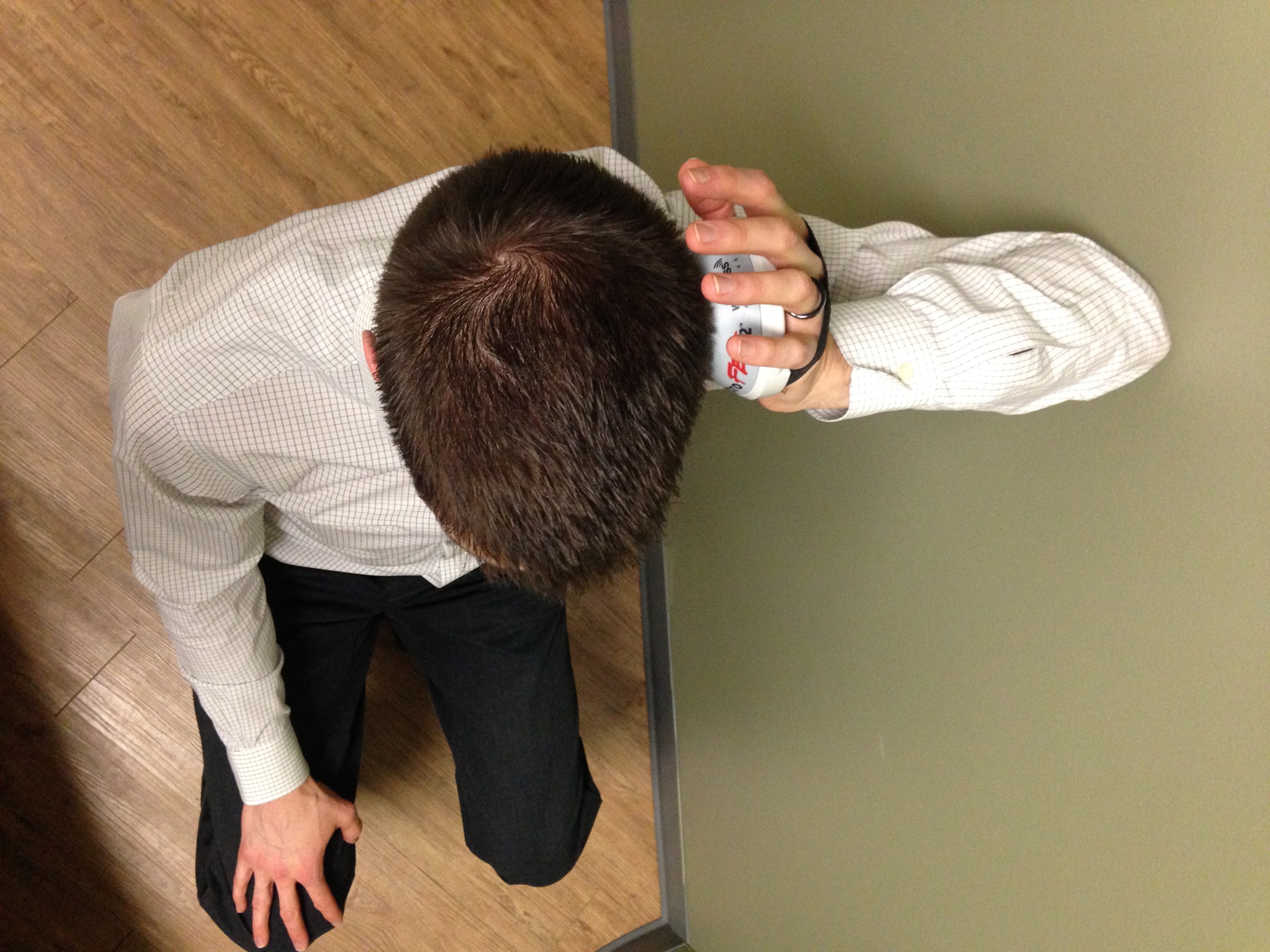


**Side Flexion**: subjects are seated with a wall on the ipsilateral side to be tested. The force pad is placed just above the ear, and the dynamometer is held by the ipsilateral hand, with the upper arm in the place of abduction, and the elbow stabilized against the wall. The distance from the wall is adjusted to ensure neutral position of the neck and trunk with elbow against the wall. “*Push your head to the right / left, as if you were trying to tip your ear down to your shoulder*.”
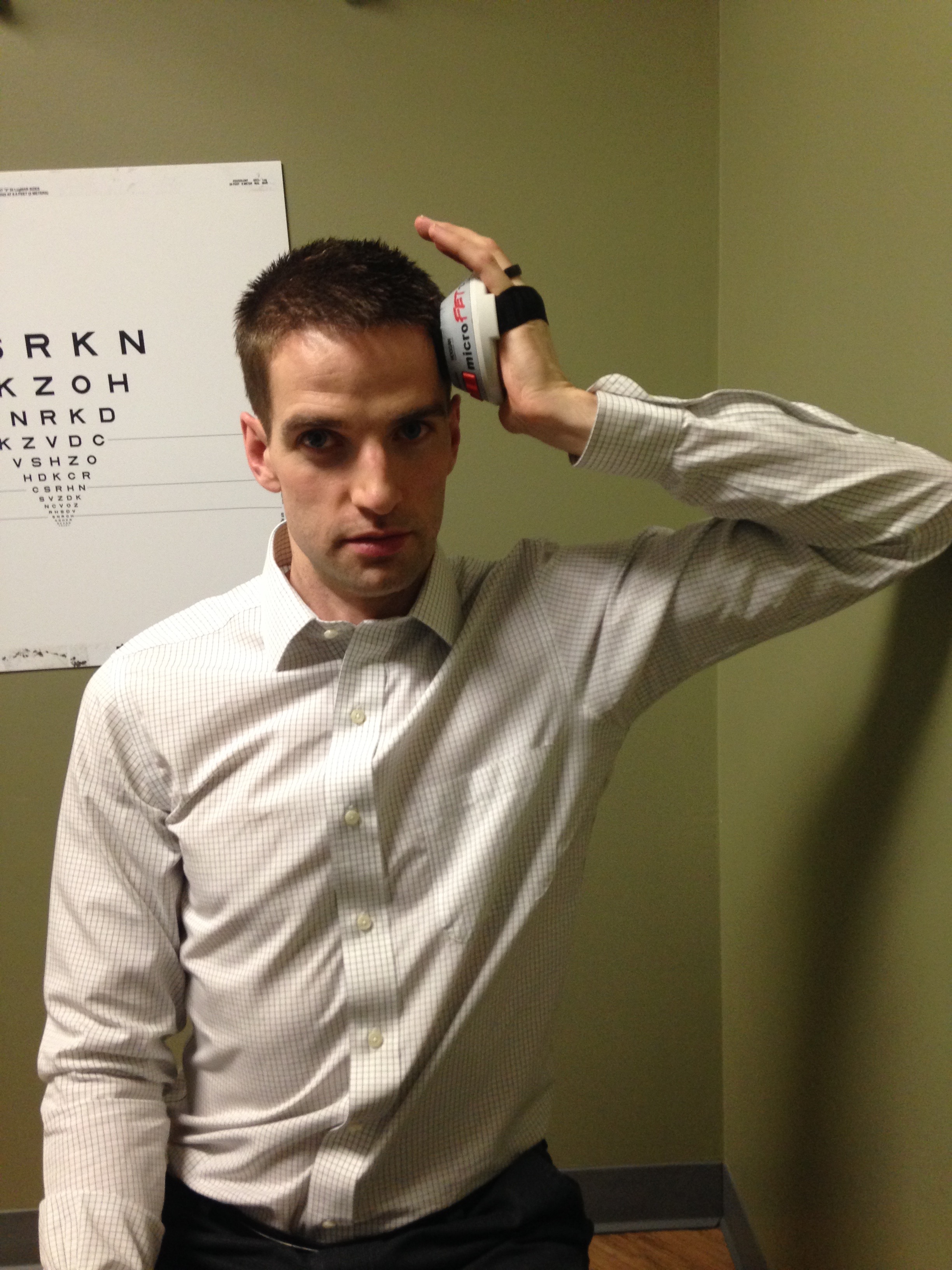


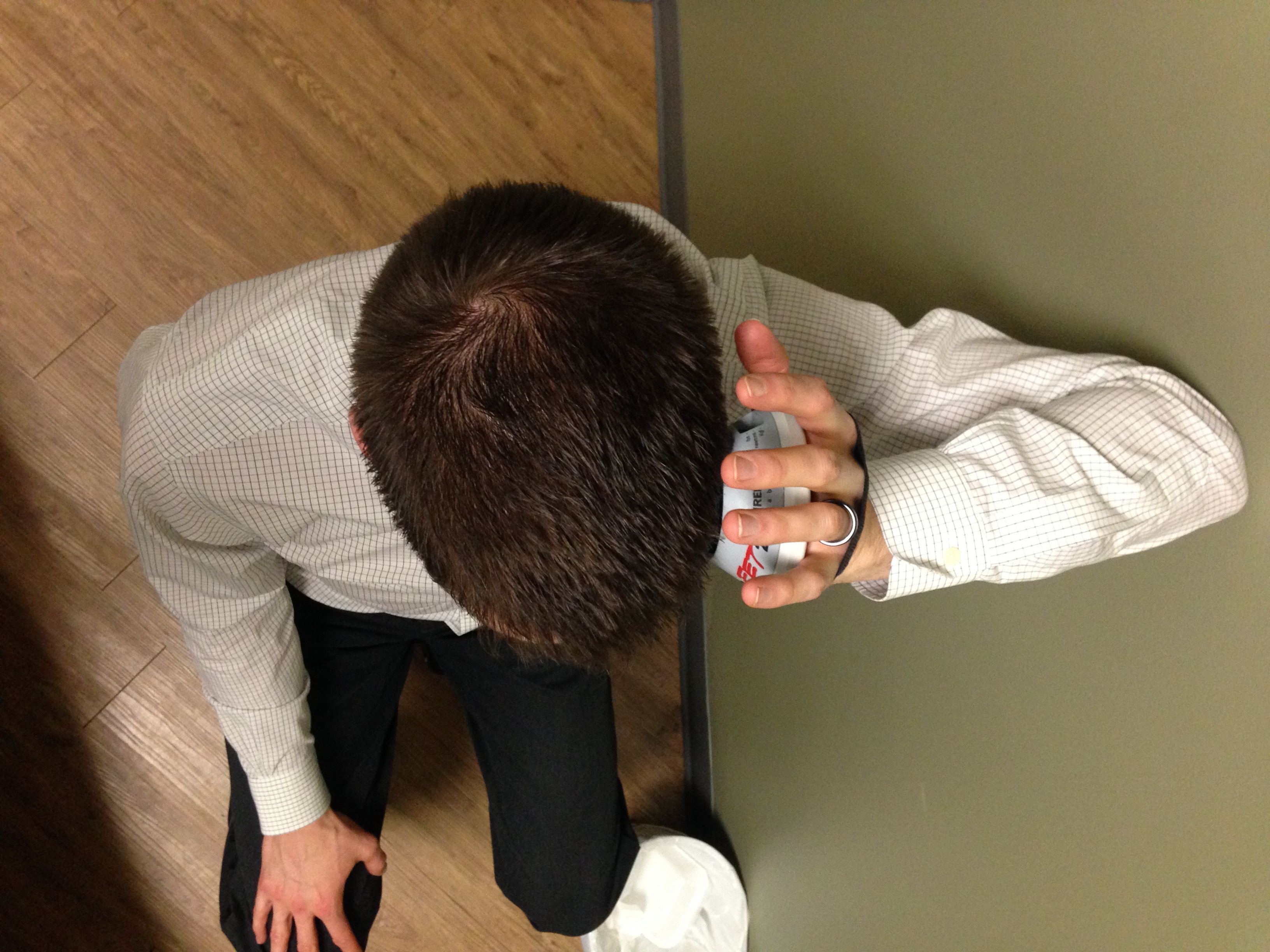


**Rotation**: subjects are seated with a wall on the ipsilateral side to be tested. The force pad is placed the temple, and the dynamometer is held by the ipsilateral hand, with the upper arm in the place of abduction, and the elbow stabilized against the wall. The distance from the wall is adjusted to ensure neutral position of the neck and trunk with elbow against the wall. “*Turn your head to the right / left, as if you were trying to turn and look over your right / left shoulder*.”

Informed consent was obtained for publication of any identifying images
