# Supplementary material for: A clinical test to assess isometric cervical strength in chronic whiplash associated disorder (WAD): a reliability study
Source: BMC Musculoskelet Disord. 2022 Aug 1;23:736. doi: 10.1186/s12891-022-05703-0 (PMC9341054; doi:10.1186/s12891-022-05703-0)
Supplement: Supplementary file 2 — Additional file 2: Appendix B. Bland-Altman plots with 95% confidence intervals of agreement. [file 12891_2022_5703_MOESM2_ESM.docx]

# Appendix B

| Bland-Altman plots with 95% confidence intervals of agreement | |
| --- | --- |
| *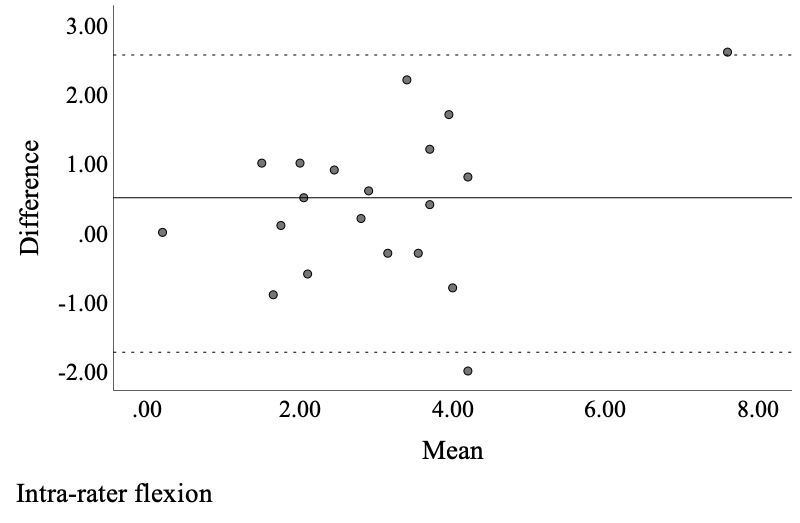* | *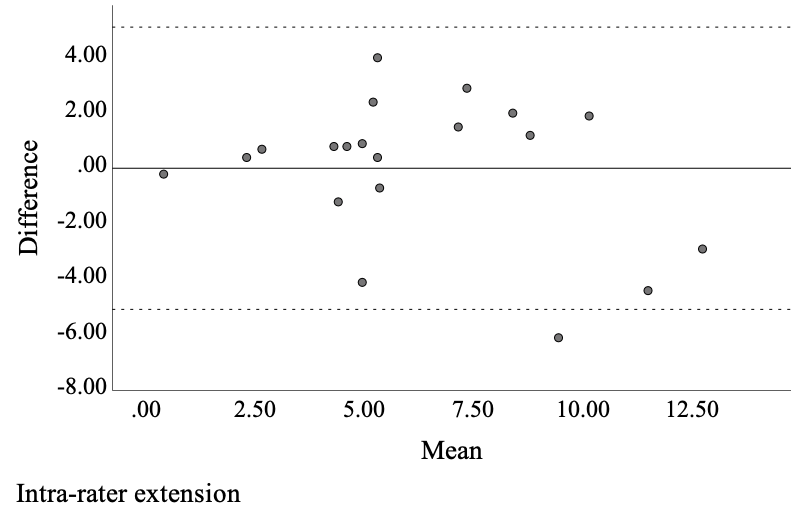* |
| *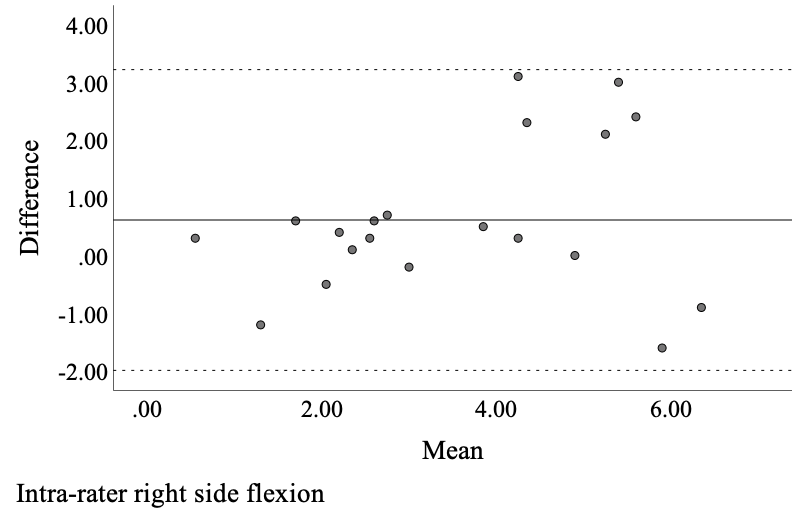* | *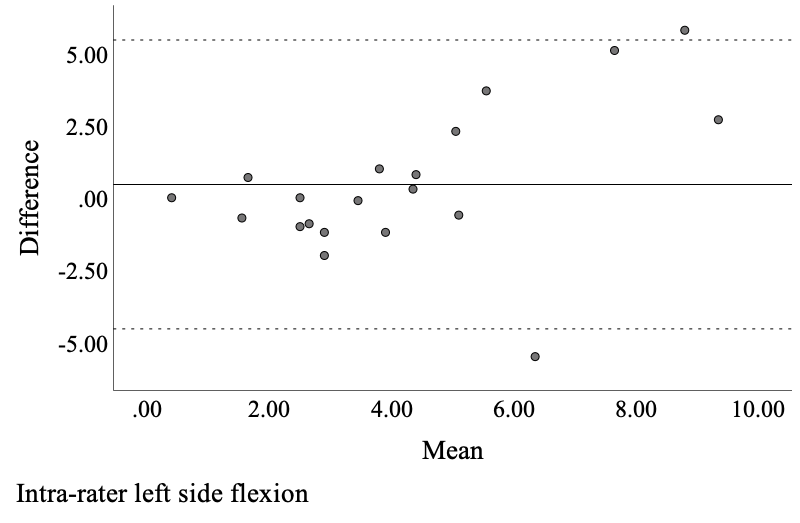* |
| *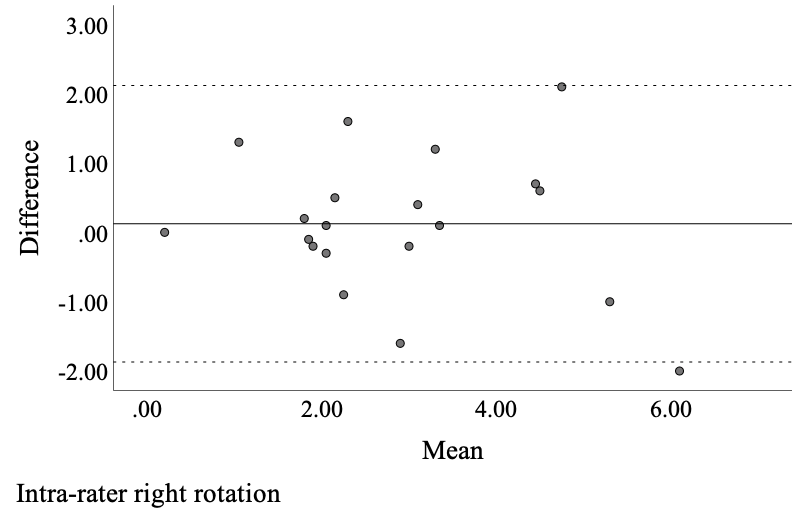* | *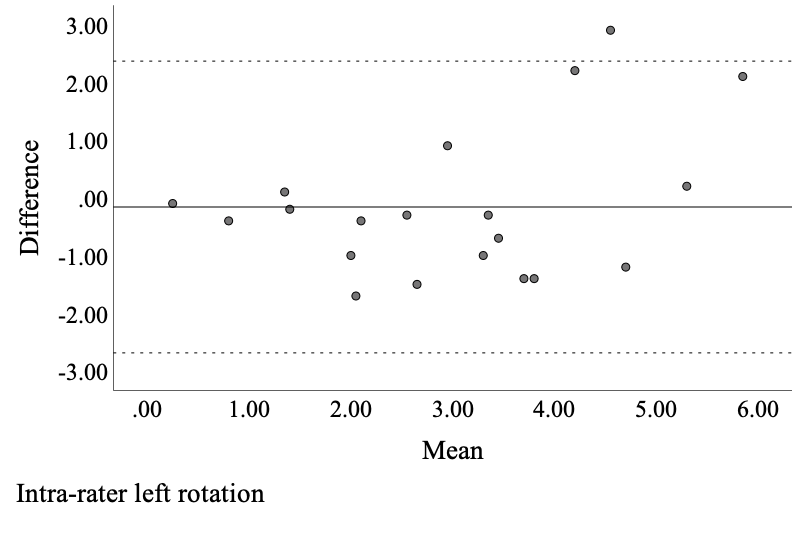* |
| *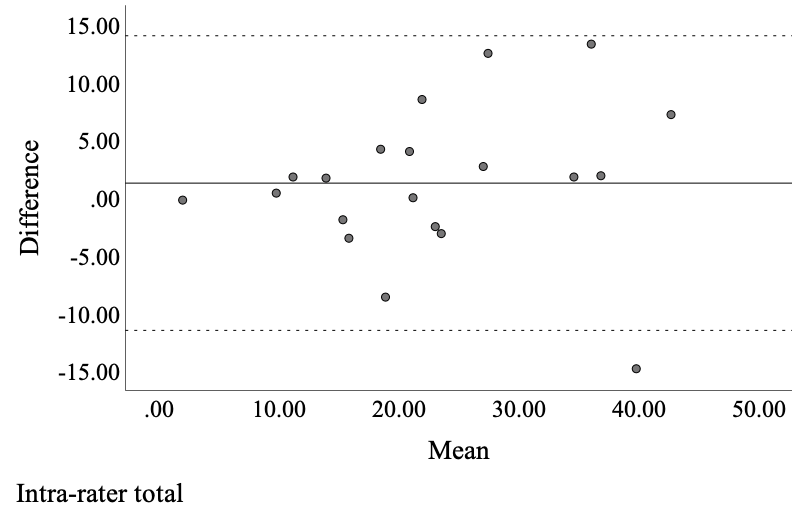* | *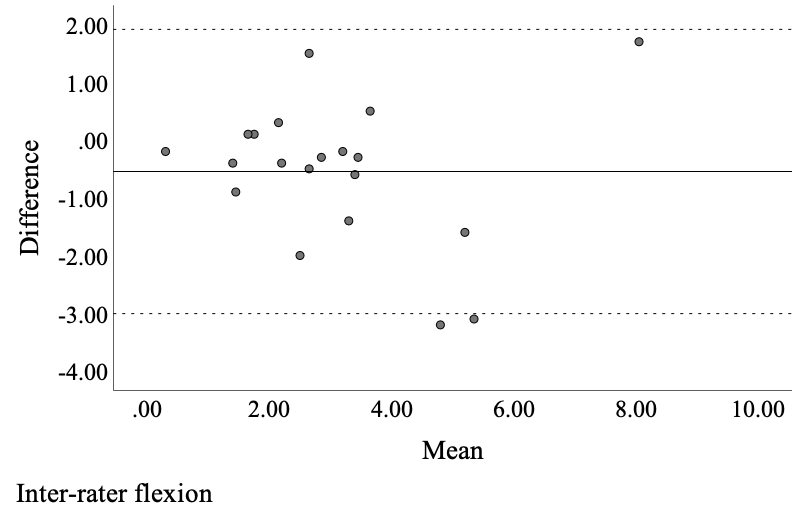* |
| *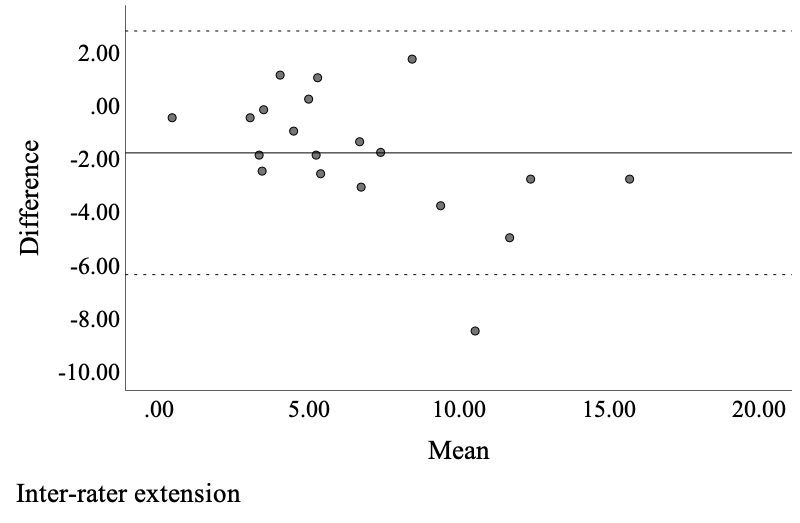* | *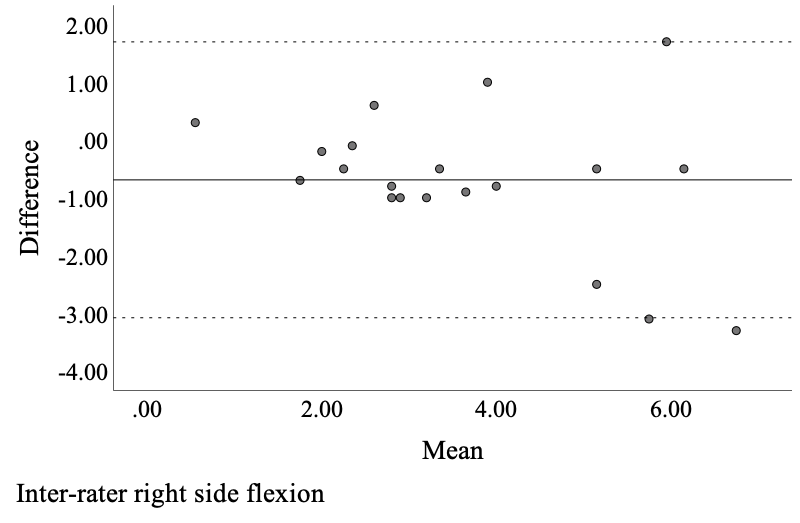* |
| *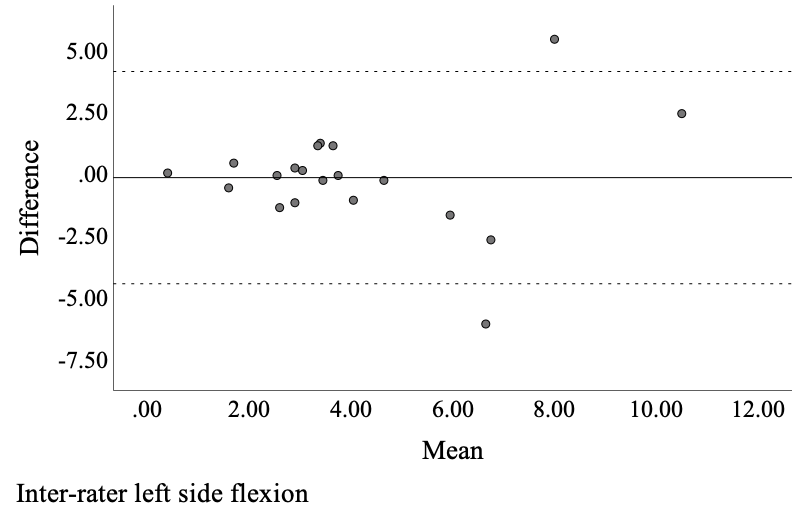* | *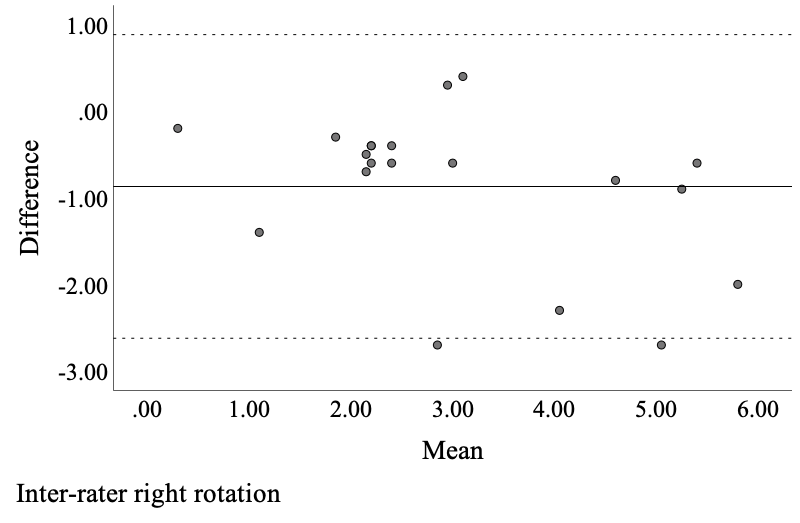* |
| *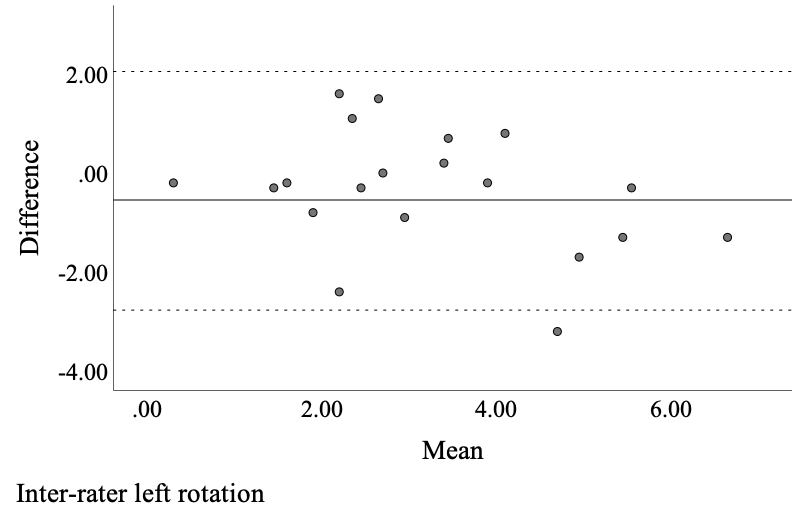* | *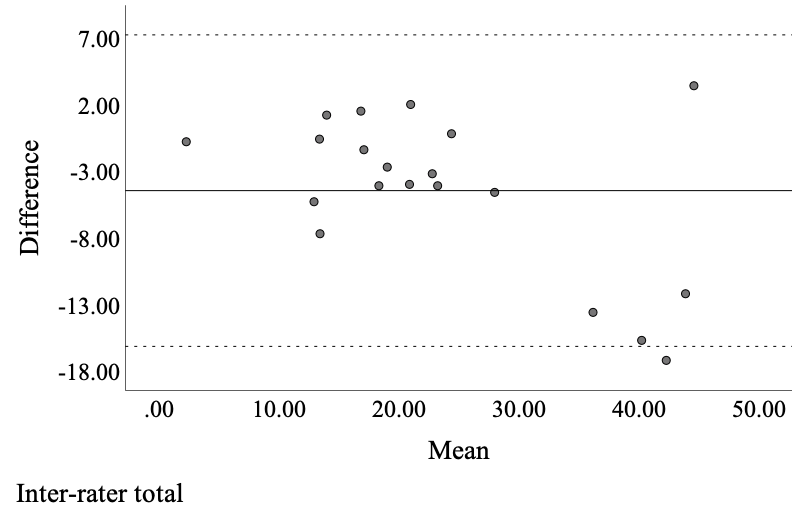* |
| Total = sum of all directional strength measures; Difference = testing session 1 minus testing session 2 strength; Mean = mean strength score of two testing sessions.  Strength scores in kgf for each testing sequence. | |
